# Non-relativistic molecular modified shifted Morse potential system

**DOI:** 10.1038/s41598-022-19179-4

**Published:** 2022-09-07

**Authors:** C. A. Onate, I. B. Okon, U. E. Vincent, E. S. Eyube, M. C. Onyeaju, E. Omugbe, G. O. Egharevba

**Affiliations:** 1 Department of Physical Sciences, Redeemer’s University, Ede, Nigeria; 2 Theoretical Physics Group, Department of Physics, University of Uyo, Uyo, Nigeria; 3 Department of Physical Sciences, Redeemer’s University, P.M.B. 230, Ede, Nigeria; 4 Department of Physics, Lancaster University, Lancaster, LA14YB UK; 5 Department of Physics, Faculty of Physical Sciences, Modibbo Adama University, Yola, Nigeria; 6 Theoretical Physics Group, Department of Physics, University of Port Harcourt, Port Harcourt, Nigeria; 7 Department of Physics, Federal University of Petroleum Resources, Effurun, Delta State Nigeria; 8 Department of Science Laboratory Technology, Delta State Polytechnic, Otefe Oghara, Nigeria

**Keywords:** Physics, Atomic and molecular physics, Chemical physics

## Abstract

A shifted Morse potential model is modified to fit the study of the vibrational energies of some molecules. Using a traditional technique/methodology, the vibrational energy and the un-normalized radial wave functions were calculated for the modified shifted Morse potential model. The condition that fits the modified potential for molecular description were deduced together with the expression for the screening parameter. The vibrational energies of SiC, NbO, CP, PH, SiF, NH and Cs_2_ molecules were computed by inserting their respective spectroscopic constants into the calculated energy equation. It was shown that the calculated results for all the molecules agreement perfectly with the experimental RKR values. The present potential performs better than Improved Morse and Morse potentials for cesium dimer. Finally, the real Morse potential model was obtained as a special case of the modified shifted potential.

## Introduction

The solutions of wave equations under different potential models are of great interest in sciences since the study of their solutions give the conceptual understanding in quantum systems. These solutions generate valuable means to check and improve models as well as numerical techniques developed to simplify complicated systems. Over the years, certain solvable techniques like Nikiforov-Uvarov method^1^, asymptotic iteration method^2–4^, proper/exact quantization rule^5–7^, 1/N shifted expansion method^8^, supersymmetric method^9–15^ factorization method^16^, formula method for bound state problems^17,18^ and others, were developed to solve the wave equations with various physical potential terms. The choice of any method depends on the nature of the problem under consideration as well as the ease in handling complicated situations that may arise. For instance, some potential terms cannot be solved in the absence of the angular momentum quantum state, hence, the solution under these potential models can be obtained by employing a suitable approximation scheme. On the other hand, certain potential models even when they admit a solution for $j = 0,$ they cannot be used to completely describe the diatomic molecules due to the absence of molecular constants like the dissociation energy, equilibrium bond separation and vibrational frequency. Therefore, some potential models that have spectroscopic constants or diatomic constants have been given much attention in the recent time. One of such potentials that possess the spectroscopic constants is the Morse potential function. The Morse potential function was proposed by Philip Morse^19^ in 1929 as a three-parameter empirical potential energy function. The Morse potential is a convenient interatomic interaction model for the potential energy of a diatomic molecule that can be use to describe interaction between an atom and a surface. This potential exists as the simplest representative of the potentials and actually results to dissociation, bringing its important over other popular potentials like Harmonic potential. The three-parameter empirical Morse potential model proposed in 1929 is given by

1 $$V(r) = D_{e} (1 - 2e^{{ - \alpha (r - r_{e} )}} + e^{{ - 2\alpha (r - r_{e} )}} ),$$

where D_e_ is the dissociation energy, r_e_ is the equilibrium bond separation and $r$ is the internuclear separation. The Morse potential given in Eq. (1), has received attentions on different molecules^20,21^. In^22,23^. The Morse potential model in Eq. (1) was reduced to the form

2 $$D_{e} (e^{{ - 2\alpha (r - r_{e} )}} - 2e^{{ - \alpha (r - r_{e} )}} ).$$

The authors obtained the ro-vibrational energy levels for hydrogen molecule at various states. According to ref.^21^, the Morse potential has been used to calculate the transition frequencies, intensities of diatomic molecules and in dynamics. The authors also pointed out that the theoretical results deduced under Morse potential deviated from the experimental data. In ref.^20^, a new form of Morse potential model

3 $$V(r) = (\ell + \beta )^{2} - (2\ell + 3)e^{ - x} + e^{ - 2x} ,$$

called shifted Morse potential was studied, where $\ell$ is a constant and $\beta$ is always one (1). This form of Morse potential model cannot be used to describe any molecule completely due to the absence of the spectroscopic parameters. Similarly, the parameters $\ell ,$ $\beta$ and $x$ lack clear physical definition in the study of molecules. The authors clearly pointed out that the three-parameter empirical Morse potential or the reduced Morse potential in Eq. (2), cannot be recovered from the Morse potential in Eq. (3) by change of variable. This state thus, draw the attention of the authors. Thus, to study any molecule under the shifted Morse potential model, it becomes expedient to construct a reparametise shifted Morse potential function that its mathematical parameters match molecular parameters. It is also necessary to condition some of the potential parameters in the shifted Morse potential such that the real Morse potential model can be retrieve. Therefore, in the present study, a dissociation energy D_e_ a constant γ are introduced. A transformation $x = \alpha (r - r_{e} ),$ is also made in Eq. (3) to have

4 $$V(r) = D_{e} \left[ {(\ell + \beta )^{2} - (2\ell + 3\gamma )e^{{ - \alpha (r - r_{e} )}} + e^{{ - 2\alpha (r - r_{e} )}} } \right].$$

From Eq. (4), the original Morse potential function can be recovered as a special case. The dissociation energy D_e_, the equilibrium bond separation r_e_ and the equilibrium harmonic vibrational frequency $\omega_{e}$ for diatomic molecules are correlated with potential energy function $V(r)$ and defined by the following relations

5 $$\left. \begin{gathered} \left. {\frac{dV(r)}{{dr}}} \right|_{{r = r_{e} }} = 0, \hfill \\ V(r \to \infty ) - V(r_{e} ) = D_{e} , \hfill \\ \left. {\frac{{d^{2} V(r)}}{{dr^{2} }}} \right|_{{r = r_{e} }} = 4\pi \mu c^{2} \omega_{e}^{2} \hfill \\ \end{gathered} \right\},$$

where $c$ is the speed of light, $\mu$ is the reduced mass,$\ell ,$ $\gamma$ and $\beta$ are connected by the following relations

6 $$\left. \begin{gathered} 3\gamma + 2\ell = 2 \hfill \\ \ell + \beta = 1 \hfill \\ \end{gathered} \right\}.$$

After some mathematical simplifications using the relations above, the parameter $\alpha$ for molecular system can be calculated by the formula

7 $$\alpha = 2\pi c\omega_{e} \sqrt {\frac{\mu }{{D_{e} (4 - 2\ell - 3\gamma )}}} .$$

The present work will study the radial Schrödinger equation under the modified shifted Morse potential model in Eq. (4) and recover the solution of the real Morse potential given in Eq. (1) from the solution of the Morse potential in Eq. (4). This study will also examine the vibrational energies of some molecules and compared with experimental RKR data as an application. To the best of our knowledge, this is the first time this potential is receiving attention. The modified shifted Morse potential (blue line) and the Morse potential (black line) are shown below.

## Parametric Nikiforov-Uvarov method

The radial Schrödinger equation for any potential model is transformed to the form^24–29^

8 $$\left[ {\frac{{d^{2} }}{{ds^{2} }} + \frac{{v_{1} - v_{2} s}}{{s(1 - v_{3} )}}\frac{d}{ds} + \frac{{ - \xi_{0} s^{2} + \xi_{1} s - \xi_{3} }}{{s^{2} (1 - v_{3} s)^{2} }}} \right]\psi (s) = 0.$$

According to Tezcan and Sever^24^, the solutions of Eq. (8) are obtained from the following conditions

9 $$v_{2} n + n(n - 1)v_{3} + v_{7} + 2v_{3} v_{8} + (2n + 1)\left( {\sqrt {v_{9} } + v_{3} \sqrt {v_{8} } - v_{5} } \right) + 2\sqrt {v_{8} v_{9} } = 0,$$

10 $$\psi (s) = Ns^{{v_{12} }} (1 - v_{3} s)^{{ - v_{12} - \frac{{v_{13} }}{{v_{3} }}}} P_{n}^{{\left( {v_{10} - 1,\frac{{v_{11} }}{{v_{3} }} - v_{10} - 1} \right)}} (1 - 2v_{3} s).$$

The values of the constants in Eqs. (9) and (10) are deduce as follows

11 $$\left. \begin{gathered} v_{4} = \frac{{1 - v_{1} }}{2},v_{5} = \frac{{v_{2} - 2v_{3} }}{2},v_{6} = v_{5}^{2} + \xi_{1} ,v_{7} = 2v_{4} v_{5} - \xi_{2} ,v_{8} = v_{4}^{2} + \xi_{3} , \hfill \\ v_{9} = v_{3} \left( {v_{7} + v_{3} v_{8} } \right) + v_{6} ,v_{10} = 1 + 2v_{4} + 2\sqrt {v_{8} } ,v_{11} = v_{2} - 2v_{5} + 2\left( {\sqrt {v_{9} } + v_{3} \sqrt {v_{8} } } \right), \hfill \\ v_{12} = v_{4} + \sqrt {v_{8} } ,v_{13} = v_{5} - \left( {\sqrt {v_{9} } + v_{3} \sqrt {v_{8} } } \right) \hfill \\ \end{gathered} \right\}.$$

According to ref.^24^, when $v_{3} = 0,$

11a $$\mathop {lim}\limits_{{v_{3} \to 0}} P_{n}^{{\left( {v_{10} - 1,\frac{{v_{11} }}{{v_{3} }} - v_{10} - 1} \right)}} (1 - v_{3} s) = L_{n}^{{v_{10} - 1}} (v_{11} s),$$

and

11b $$\mathop {lim}\limits_{{v_{3} \to 0}} (1 - v_{3} s)^{{ - v_{12} - \frac{{v_{13} }}{{v_{3} }}}} = e^{{v_{13} s}} .$$

Following Eqs. (11a) and (11b), Eq. (10) reduces to

12 $$\psi (s) = Ns^{{v_{12} }} e^{{v_{13} s}} L_{n}^{{(v_{10} - 1)}} (v_{11} s).$$

### Bound state solutions

The radial Schrödinger equation for any given potential model of interest is given by^30–38^

13 $$\left[ { - \frac{{\hbar^{2} }}{2\mu }\frac{{d^{2} }}{{dr^{2} }} + V(r) - E_{v,j} + \frac{{\hbar^{2} }}{2\mu }\frac{j(j + 1)}{{r^{2} }}} \right]R_{v,j} (r) = 0,$$

where $\hbar$ stands for reduced Planck’s constant,$v$ is vibrational quantum state, j is the vibrational angular momentum quantum state, $V(r)$ is the potential, $E_{v,j}$ is the energy and $R_{v,j} (r)$ is the wave function. The term $r^{ - 2}$ in Eq. (13) can be approximated by the formula

14 $$\frac{1}{{r^{2} }} \approx \frac{{d_{0} + d_{1} e^{ - \alpha r} + d_{2} e^{ - 2\alpha r} }}{{r_{e}^{2} }},$$

where

15 $$\left. {d_{0} = 1 + \frac{3}{{\alpha r_{e} }}\left( {\frac{1}{{\alpha r_{e} }} - 1} \right),d_{1} = \frac{{2e^{{\alpha r_{e} }} }}{{\alpha r_{e} }}\left( {2 - \frac{3}{{\alpha r_{e} }}} \right),d_{3} = \frac{{e^{{2\alpha r_{e} }} }}{{\alpha r_{e} }}\left( {\frac{3}{{\alpha r_{e} }} - 1} \right)} \right\}.$$

Substituting Eqs. (4) and (14) into Eq. (13) and invoking $y = e^{ - \alpha r} ,$ we obtain the following

16 $$\left[ {\frac{{d^{2} }}{{dy^{2} }} + \frac{1}{y}\frac{d}{dy} + \frac{{ - \left( {\lambda e^{{2\alpha r_{e} }} + Jd_{2} } \right)y^{2} + \left( {\lambda (2\ell + 3\gamma )e^{{\alpha r_{e} }} + Jd_{1} } \right) - \lambda (\ell + \beta )^{2} - Jd_{0} + \frac{{2\mu E_{v,j} }}{{\alpha^{2} \hbar^{2} }}}}{{y^{2} }}} \right]R_{v,j} (y) = 0,$$

17 $$\lambda = \frac{{2\mu D_{e} }}{{\alpha^{2} \hbar^{2} }},J = \frac{j(j + 1)}{{\alpha^{2} r_{e}^{2} }}.$$

Relating Eq. (16) with Eq. (8), we then obtain the parameters in Eq. (11) as follows

18 $$\left. \begin{gathered} v_{1} = 1,v_{2} = v_{3} = v_{4} = v_{5} = 0,v_{6} = \lambda e^{{2\alpha r_{e} }} + Jd_{2} ,v_{7} = Jd_{1} - \lambda (2\ell + 3\gamma )e^{{\alpha r_{e} }} , \hfill \\ v_{8} = \lambda (\ell + 1)^{2} + Jd_{0} - \frac{{\lambda E_{v,j} }}{{D_{e} }},v_{9} = \lambda e^{{2\alpha r_{e} }} + Jd_{2} ,v_{10} = 1 + 2\sqrt {\lambda (\ell + \beta )^{2} + Jd_{0} - \frac{{\lambda E_{v,j} }}{{D_{e} }}} , \hfill \\ v_{11} = 2\sqrt {\lambda e^{{2\alpha r_{e} }} + Jd_{2} } ,v_{12} \sqrt {\lambda (\ell + \beta )^{2} + Jd_{0} - \frac{{\lambda E_{v,j} }}{{D_{e} }}} ,v_{13} = - \sqrt {\lambda e^{{2\alpha r_{e} }} + Jd_{2} } \hfill \\ \end{gathered} \right\}.$$

Plugging Eq. (18) into Eqs. (9) and (12), the non-relativistic energy equation and its unnormailized wave function are obtain as

19 $$E_{v,j} = D_{e} (\ell + \beta )^{2} + \frac{{\alpha^{2} \hbar^{2} }}{2\mu }\left[ {Jd_{0} - \left( {\frac{{\frac{{\mu D_{e} (2\ell + 3\gamma )e^{{\alpha r_{e} }} }}{{\alpha^{2} \hbar^{2} }} - Jd_{1} - \left( {v + \frac{1}{2}} \right)\sqrt {Jd_{2} + \lambda e^{{2\alpha r_{e} }} } }}{{\sqrt {Jd_{2} + \lambda e^{{2\alpha r_{e} }} } }}} \right)^{2} } \right],$$

20 $$R_{v,j} (y) = Ny^{{\sqrt {\lambda (\ell + 1)^{2} + Jd_{0} - \frac{{\lambda E_{v,j} }}{{D_{e} }}} }} e^{{ - y\sqrt {\lambda e^{{\alpha r_{e} + Jd_{2} }} } }} L_{n}^{{2\sqrt {\lambda (\ell + 1)^{2} + Jd_{0} - \frac{{\lambda E_{v,j} }}{{D_{e} }}} }} \left( {2\sqrt {\lambda e^{{\alpha r_{e} + Jd_{2} }} } y} \right).$$

## Discussion

**Figure 1:**
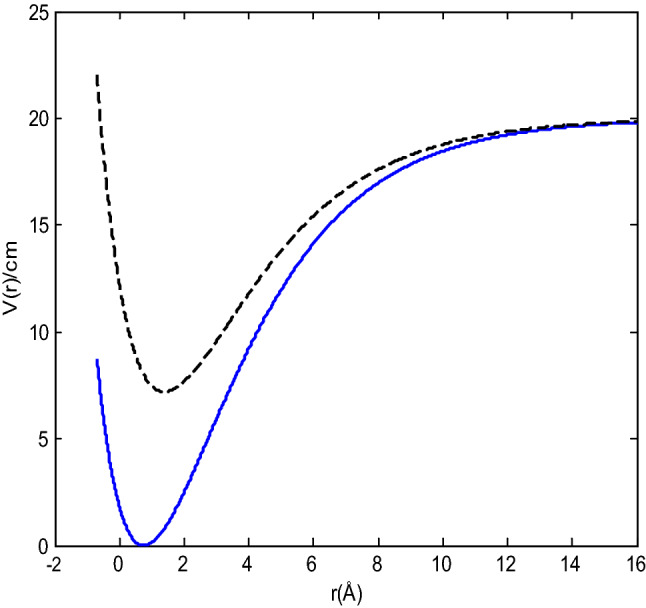
Shifted Morse potential and Morse potential with α = 0.35 cm^−1^ $r_{e} = 0.75$ Å, D_e_ = 20 cm^−1^ $\gamma = 0.3,$ $\beta = 0.65,$ and $\ell = 0.35$.

The presentation of the modified shifted Morse and Morse potentials are shown in Fig. 1. It can be seen that the modified shifted Morse potential and the Morse potential coincide as $r$ increases. However, for $r < 5$ Å, the two potentials have little discrepancy but have the same shape. The variation could be probably due to the effects of the non-molecular parameters in the shifted Morse potential.

Table 1 shows the values of some molecular constants. By imputing these molecular constants into Eq. (7), the value of the potential parameter $\alpha$ for each molecule is calculated. All the spectroscopic constants except the Cs_2_, are obtained from ref.^41^. The spectroscopic constants for Cs_2_ molecule are obtained from ref.^39^. The theoretical values for pure vibrational energies for $X^{1} \prod$ state of SiC, $X^{4} \sum$ state of NbO, $X^{2} \prod$ state of PH, $X^{2} \prod$ state of NH, $X^{1} \sum^{ + }$ state of SiF, $X^{2} \sum^{ + }$ state of CP and $X^{1} \sum_{g}^{ + }$ state of Cs_2_ are obtained using Eq. (19). Tables 2, 3, 4, 5 and 6 respectively contained the energies of vibrational levels for different molecules. These tables showed the cmparison of the experimental data and the theoretical values for the different molecules listed in Table 1. The numerical values for these molecules are obtained using MATLAB 7.5.0 software. The calculated results are found to be in good agreement with the experimental RKR values.

**Table 1:** Spectroscopic constants used for the study^39,40^.

Molecule	D_e_ (cm^−1^)	$\omega_{e}$ (cm^−1^)	$r_{e}$ (Å)
SiC $(X^{3} \prod )$	27,336.85	954.20	1.7320
NbO $(X^{4} \sum )$	50,032.53	989.00	1.6909
PH $(X^{2} \prod )$	27,683.75	2382.75	1.4247
NH $(X^{2} \prod )$	26,281.82	3047.58	1.0692
SiF $(X^{1} \sum^{ + } )$	43,982.17	1050.37	1.5265
CP $(X^{2} \sum^{ + } )$	44,092.79	1239.80	1.5619
Cs_2_ $(X^{1} \sum_{g}^{ + } )$	3649.50	42.020	4.6480

**Table 2:** Contains the calculated energy of vibrational levels (cm^−1^) and experimental data (cm^−1^) of SiC and NbO molecules for the modified shifted Morse potential function.

$v$	SiC $(X^{3} \prod )$			NbO $(X^{4} \sum )$		
	RKR^40^	Calculated	LTE			
0	475.47	475.02	0.45	493.43	493.28	0.15
1	1416.67	1412.57	4.10	1474.88	1472.50	2.38
2	2344.87	2333.46	11.41	2448.56	2441.95	6.61
3	3260.07	3237.70	22.37	3414.58	3401.63	12.95
4	4162.27	4125.29	36.98	4372.94	4351.53	21.41
5	5051.47	4996.22	55.25	5323.64	5291.66	31.98
6	5927.67	5850.50	77.17	6266.68	6222.01	44.67
7	6790.67	6688.13	102.54	7202.06	7142.58	59.48

**Table 3:** Contains the calculated energy of vibrational levels (cm^−1^) and experimental data (cm^−1^) of PH and NH molecules for the modified shifted Morse potential function.

$v$	PH $(X^{2} \prod )$			NH $(X^{2} \prod )$		
	RKR^40^	Calculated	LTE	RKR^40^	Calculated	LTE
0	1180.95	1178.56	2.39	1505.74	1501.70	4.04
1	3480.36	3458.77	21.59	4408.94	4372.59	36.35
2	5696.43	5636.43	60.00	7167.76	7066.78	100.98
3	7829.16	7711.55	117.61	9782.20	9584.27	197.93
4	9878.55	9684.14	194.41	12,252.26	11,925.07	327.19
5	11,844.61	11,554.18	290.43	14,577.94	14,089.17	488.77

**Table 4:** Contains the calculated energy of vibrational levels (cm^−1^) and experimental data (cm^−1^) of SiF and CP molecules for the modified shifted Morse potential function.

$v$	SiF $(X^{1} \sum^{ + } )$			CP $(X^{2} \sum^{ + } )$		
	RKR^40^	Calculated	LTE	RKR^40^	Calculated	LTE
0	523.95	523.62	0.33	618.19	617.72	0.47
1	1564.43	1561.44	2.99	1844.32	1840.09	4.23
2	2595.02	2586.73	8.29	3056.76	3045.03	11.73
3	3615.72	3599.47	16.25	4255.53	4232.54	22.99
4	4626.53	4599.67	26.86	5440.62	5402.62	38.00
5	5627.44	5587.33	40.11

**Table 5:** Contains the calculated energy of vibrational levels (cm^−1^) and experimental data (cm^−1^) of cesium dimer for the modified shifted Morse and Morse potential models.

*v*	RKR	^41^	$\ell = 1$	$\ell = 0$	$\ell = - 1$
0	14.4248	14.42670	14.42670	14.42670	14.42670
1	43.1680	43.16520	43.16520	43.16520	43.16520
2	71.7657	71.75040	71.75040	71.75040	71.75040
3	100.2211	100.1822	100.1822	100.1822	100.1822
4	128.5375	128.4608	128.4608	128.4608	128.4608
5	156.7182	156.5860	156.5860	156.5860	156.5860
6	184.7663	184.5579	184.5579	184.5579	184.5579
7	212.6851	212.3765	212.3765	212.3765	212.3765
8	240.4778	240.0418	240.0418	240.0418	240.0418
9	268.1477	267.5537	267.5537	267.5537	267.5537
10	295.6980	294.9123	294.9124	294.9124	294.9124
11	323.1320	323.1177	323.1177	323.1177	323.1177
12	350.4529	349.1697	349.1697	349.1697	349.1697

**Table 6:** Contains the calculated energy of vibrational levels (cm^−1^) and experimental values (cm^−1^) of nitrogen dimer.

	RKR	^42^	Present
0	1184.4539	1174.9477	1174.9477
1	3526.3576	3498.7289	3498.7289
2	5833.4516	5787.6913	5787.6914
3	8107.0460	8041.8351	8041.8351
4	10,348.312	10,261.160	10,261.160
5	12,558.287	12,445.666	12,445.666
6	14,737.876	14,595.353	14,595.354
7	16,887.859	16,710.222	16,710.222
8	19,008.895	18,790.272	18,790.272
9	21,101.519	20,835.503	20,835.503

To determine the fitting excellence of the shifted Morse potential function, the average absolute percentage deviation for each molecule is calculated using the formula

21 $$\sigma_{a} = \frac{100}{N}\sum \left( {\frac{{E_{R} - C_{R} }}{{E_{R} }}} \right),$$

where $E_{R}$ is the experimental data, $C_{R}$ is the calculated values and $N$ is the number of observation. Following the formula given in Eq. (21), the average absolute percentage deviation for the molecules studied are calculated to be; 0.8234% for SiC, 0.0724% for NbO, 0.2867% for PH, 0.3876% for NH, 0.0852% for SiF, 0.1018% for CP and 0.0138% for Cs_2_. As it can be seen, the average absolute percentage deviation for each of the molecules is less than unity. This shows that the calculated values are in good agreement with the experimental data. It has been observed that the experimental date are greater than the calculated values for all the molecules studied. The computation of the results also revealed that the LTE (the disparity between the experimental data and calculated values at each vibration state) for each molecule increases with the vibrational quantum state. It is noted that the higher the vibrational energies of the molecule, the higher the LTE. Our results also showed that the minimum LTE for each molecule is obtained at the lowest vibrational quantum level. To deduce more fitness of the modified shifted Morse potential to the study of molecules, the average deviation for cesium dimer is calculated in terms of the dissociation energy and compared with the results in ref.^42^. In the present study, the absolute deviation for the Cs_2_ is 0.0049% of the observed value while in ref.^24^, it was 0.036% of D_e_ and 0.121% of D_e_ for improved Rosen-Morse potential and Morse potential respectively. Thus, the modified shifted Morse performs better than the improved Rosen-Morse potential and Morse potential for cesium dimer.

To assertain the validity of the condition given in Eq. (6), the result for Cs_2_ is computed for $\ell = - 1,$ $\ell = 0$ and $\ell = 1.$ It is observed that the result for the three values of $\ell$ are the same. This simply shows that the condition given in Eq. (3) justify the fitness of the shifted Morse potential for the representation of molecules. The comparison of the calculated results of cesium dimer for the Morse potential and the results for modified shifted Morse are presented Table 5. The calculated results of cesium dimer for the Morse potential and modified shifted Morse potential are almost the same except for the 10th vibrational quantum state where the result for the modified shifted Morse potential is closer to the RKR data by 0.001 cm^−1^. The comparison of the calculated values of nitrogen dimer for Morse potential and modified shifted Morse potential are presented in Table 6 for ten different vibrational quantum states. The calculated results for the two potential models agreed with the RKR data. The calculated results for the two potential models are almost the same except for the second and sixth vibrational states where the results for modified shifted Morse potential are closer to the RKR data. The result for the second vibrational quantum state is 0.0001 cm^−1^ closer to the RKR data while at the sixth vibrational quantum state, it is 0.001 cm^−1^ closer to the RKR data.

The Shifted Morse potential function in this study has Morse potential function given in Eqs. (1) and (2) respectively as its special cases. When $\ell = \beta = 0,$ and $\gamma = 2/3$ the Morse potential given in Eq. (2) is obtained

22 $$V(r) = D_{e} \left[ { - 2e^{{ - \alpha (r - r_{e} )}} + e^{{ - 2\alpha (r - r_{e} )}} } \right],$$

and the energy Eq. (16) becomes

23 $$E_{v,j} = \frac{{\alpha^{2} \hbar^{2} }}{2\mu }\left[ {Jd_{0} - \left( {\frac{{\frac{{2\mu D_{e} e^{{\alpha r_{e} }} }}{{\alpha^{2} \hbar^{2} }} - Jd_{1} - \left( {v + \frac{1}{2}} \right)\sqrt {Jd_{2} + \lambda e^{{2\alpha r_{e} }} } }}{{\sqrt {Jd_{2} + \lambda e^{{2\alpha r_{e} }} } }}} \right)^{2} } \right].$$

The result of the vibrational energy equation in Eq. (23) do not agree with the results obtained using Eq. (16). This is due to the exclusion of the first dissociation energy in the potential. When $\ell = 0,$ $\gamma = 2/3$ and $\beta = 1,$ the Morse potential given in Eq. (1) is obtained

24 $$V(r) = D_{e} \left[ {1 - 2e^{{ - \alpha (r - r_{e} )}} + e^{{ - 2\alpha (r - r_{e} )}} } \right],$$

and the energy equation of Eq. (16) turns to

25 $$E_{v,j} = D_{e} + \frac{{\alpha^{2} \hbar^{2} }}{2\mu }\left[ {Jd_{0} - \left( {\frac{{\frac{{2\mu D_{e} e^{{\alpha r_{e} }} }}{{\alpha^{2} \hbar^{2} }} - Jd_{1} - \left( {v + \frac{1}{2}} \right)\sqrt {Jd_{2} + \lambda e^{{2\alpha r_{e} }} } }}{{\sqrt {Jd_{2} + \lambda e^{{2\alpha r_{e} }} } }}} \right)^{2} } \right].$$

The results of Eqs. (16) and (25) perfectly aligned with each other.

## Conclusion

The solution for modified shifted Morse potential model was obtained for any $j -$ state. The energy eigenvalues of some molecules were numerically obtained for the modified shifted Morse potential. The calculated results for all the molecules agreed with the experimental RKR values. It was observed that changing the value of $\ell$ has no effect on the numerical result provided the conditions given in Eq. (6) are obeyed. The original Morse potential model was recovered from the modified shifted Morse potential model. It is shown that the modified shifted Morse potential performs better than the improved Rosen-Morse and Morse potentials for cesium dimer.

## Data Availability

All data generated or analysed during this study are included in these published articles^39–41^.
